# TMAO is Associated with Mortality: Impact of Modestly Impaired Renal Function

**DOI:** 10.1038/s41598-017-13739-9

**Published:** 2017-10-23

**Authors:** Eke G. Gruppen, Erwin Garcia, Margery A. Connelly, Elias J. Jeyarajah, James D. Otvos, Stephan J. L. Bakker, Robin P. F. Dullaart

**Affiliations:** 1 Department of Nephrology, University of Groningen and University Medical Center Groningen, Groningen, The Netherlands; 2 Department of Endocrinology, University of Groningen and University Medical Center Groningen, Groningen, The Netherlands; 3 Laboratory Corporation of America Holdings (LabCorp), Morrisville, NC USA

## Abstract

Trimethylamine-N-Oxide (TMAO) is a microbiome-related metabolite that is cleared by the kidney and linked to renal function. We explored the relationship between TMAO and all-cause mortality, and determined whether this association was modified by renal function. A prospective study was performed among PREVEND participants to examine associations of plasma TMAO with all-cause mortality. After median follow-up of 8.3 years in 5,469 participants, 322 subjects died. TMAO was positively associated with age, body mass index, type 2 diabetes mellitus and inversely with estimated glomerular filtration rate (eGFR*creatcysC*)(all P < 0.001). Subjects in the highest versus lowest TMAO quartile had a crude 1.86-fold higher mortality risk (P_trend_ < 0.001). After adjustment for several risk factors, TMAO remained associated with all-cause mortality [HR:1.36 (95% CI, 0.97–1.91),P_trend_ = 0.016]. This association was lost after further adjustment for urinary albumin excretion and eGFR [HR:1.15 (95% CI, 0.81–1.64),P_trend_ = 0.22]. The association of TMAO with mortality was modified by eGFR in crude and age- and sex-adjusted analyses (interaction P = 0.002). When participants were stratified by renal function (eGFR < vs. ≥90 mL/min/1.73 m^2^), TMAO was associated with all-cause mortality only in subjects with eGFR <90 mL/min/1.73 m^2^ [adjusted HR:1.18 (95% CI, 1.02–1.36),P = 0.023]. In conclusion, TMAO is associated with all**-**cause mortality, particularly in subjects with eGFR <90 mL/min/1.73 m^2^.

## Introduction

Trimethylamine-N-Oxide (TMAO) is a metabolite that is produced from trimethylamine containing nutrients, such as carnitine, phosphatidylcholine and choline, which are abundant in a Western diet^1–14^. In animal models, TMAO plays a pathogenic role in the development of atherosclerosis^2^. Clinically, TMAO is increasingly recognized as an important pathophysiological metabolite largely due to its link with adverse cardiovascular (CV) outcomes, especially in subjects at high CV risk^2–6,8–14^. In addition to its association with primary CV events, TMAO has been shown to be useful in secondary risk stratification and as a prognostic marker in patients with acute coronary syndrome^15,16^.

TMAO is cleared by the kidney, is linked to impaired renal function and may be related to increased CV risk in patients with chronic kidney disease (CKD)^17–21^. Early interrogation of the potential effects of dietary manipulation in mice, mimicking long-term exposure to elevated TMAO, revealed that TMAO may contribute to progressive renal fibrosis and renal function impairment^18^. TMAO levels have been shown to be elevated in subjects with advanced CKD^17–21^. However, equivocal associations of TMAO with renal function and all-cause mortality have been reported in subjects with late-stage CKD. Two recent studies demonstrated that serum TMAO predicted all-cause mortality in CKD patients who were candidates for renal transplantation^18,19^, while a third study reported that TMAO levels were elevated, but not predictive of all-cause mortality in patients new to hemodialysis^22^. All together, these findings make it relevant to test the extent to which CKD may modify the association of TMAO with mortality in the general population.

The aims of this study were i) to explore the relationship between TMAO and all-cause mortality in a large, well-characterized population-dwelling cohort and ii) to determine whether this association is modified by renal function impairment. To this end we prospectively explored the association of circulating TMAO with mortality in the Prevention of Renal and Vascular End-Stage Disease (PREVEND) study, which focuses on the impact of renal function and albuminuria on cardiometabolic disorders.

## Materials and Methods

### Study design and population

Details of the PREVEND study are described elsewhere^23–25^, In summary, in 1997 through 1998, all inhabitants of the city of Groningen, The Netherlands, between the ages of 28 and 75 years (85,421 subjects) were asked to send in a morning urine sample and to fill out a short questionnaire. Pregnant women and subjects with type 1 diabetes mellitus were excluded. The urinary albumin concentration was assessed in 40,856 responders. Subjects with a urinary albumin concentration ≥10 mg/L (n = 7,768) were invited to participate, of whom 6,000 were enrolled. In addition, a randomly selected group with a urinary albumin concentration of <10 mg/L (n = 3,394) was invited to participate in the cohort, of whom 2,592 were enrolled. These 8,592 individuals constitute the initial PREVEND cohort. The second screening took place from 2001 through 2003 (n = 6,894), which was the starting point of the present evaluation. The PREVEND study has been approved by the medical ethics committee of the University Medical Center Groningen, and was performed according to the principles outlined in the Declaration of Helsinki. All participants provided written informed consent. For the present study, subjects with missing values of TMAO at baseline were excluded, leaving 5,469 subjects for the analysis (Supplemental Fig. 1).

### Follow-up and outcome definitions

Follow-up time was defined as the period between the second screening round (baseline) and events defined as death, loss to follow-up, or the end of follow up time (01-01-2011), whichever came first. If a person had moved to an unknown destination, the date on which the person was dropped from the municipal registry was used as the census date. Data on mortality were obtained from the municipal register, and the cause of death was obtained by linking the number of the death certificate to the primary cause of death as coded by a physician from the Central Bureau of Statistics. All data were coded according to the International Classification of Diseases. The Ninth Revision (ICD-9) was used for data until 01-01-2009; after this date, data were coded according to the Tenth Revision (ICD-10). CVD was defined as the combined endpoint of incident cardiovascular morbidity and mortality which includes the following events: acute myocardial infarction, acute and subacute ischaemic heart disease, occlusion or stenosis of the precerebral or cerebral arteries or the following procedures: coronary artery bypass grafting, percutaneous transluminal coronary angioplasty or other vascular interventions (i.e. percutaneous transluminal angioplasty or bypass grafting of the aorta and peripheral vessels)^26^.

### Baseline measurements and definitions

Body mass index (BMI) was calculated as weight (kg) divided by height squared (meter^2^). Smoking status was categorized as never, former and current. Blood pressure was measured with an automatic Dinamap XL Model 9300 series device (Johnson-Johnson Medical, Tampa, FL, USA). Hypertension was defined as a systolic blood pressure (SBP) >140 mmHg or a diastolic blood pressure (DPB) >90 mmHg, and/or the use of anti-hypertensive drugs. T2DM was defined as a fasting serum glucose level >7.0 mmol/L, a non-fasting plasma glucose level >11.1 mmol/L, self-report of a physician diagnosis and/or the use of glucose lowering drugs, retrieved from a central pharmacy registry^27^. Estimated glomerular filtration rate (eGFR*creat-cysC*) was calculated using the combined creatinine cystatin C-based CKD Epidemiology Collaboration equation from 2012^27^. eGFR*creat-cysC* < and ≥90 mL/min/1.73 m^2^ was used to stratify subjects according to renal function. The urinary albumin concentration was multiplied by urine volume to obtain a value in mg per 24 h. The two 24-h urinary albumin values of each subject per examination were averaged.

### Laboratory measurements

Subjects were requested to refrain from eating and drinking during 8 hours prior to their visit in the outpatient clinic (between 8:00 a. um and 1:00 pm). Blood samples were provided and stored at −80 °C. Nuclear magnetic resonance (NMR) spectra were collected from EDTA plasma samples using the Vantera Clinical Analyzer^28^. TMAO was quantified from one-dimensional (1D) proton (^1^H) Carr-Purcell-Meiboom-Gill (CPMG) spectra using a deconvolution assay as previously described^29^. The TMAO assay has intra- and inter-assay coefficients of variation (CV%) of 4.3 and 9.8%, respectively, and a limit of quantitation of 3.3 µM. Plasma glucose was measured as described^30,31^. Serum total cholesterol was assayed on an automatic analyzer type MEGA (Merck, Darmstadt, Germany) using the CHOD-PAP-method. Triglycerides (TG) and high density lipoprotein cholesterol (HDL-C) were measured on a Beckman Coulter AU Analyzer (Beckman Coulter, Brea, CA). LDL-C was calculated using the Friedewald formula in case of triglycerides <4.5 mmol/L. Non-HDL-C was calculated as the difference between TC and HDL-C. Measurement of serum creatinine was performed by an enzymatic method on a Roche Modular analyzer (Roche Diagnostics, Mannheim, Germany). Serum cystatin C concentrations were measured by Gentian Cystatin C Immunoassay (Gentian AS, Moss, Norway) on a Modular analyzer (Roche Diagnostics). Urinary albumin excretion (UAE) was measured by nephelometry with a threshold of 2.3 mg/L, and intra- and inter-assay CV% of 2.2% and 2.6%, respectively, (Dade Behring Diagnostic, Marburg, Germany).

### Statistical analysis

Statistical analyses were performed using statistical software SPSS version 22.0 (SPSS Inc, Chicago, IL) and STATA version 13.1 (StataCorp, College Station, TX: StataCorp LP). Normally distributed data were expressed as mean ± SD and skewed data as median [interquartile range]. Skewed data were normalized by logarithmic transformation before analyses, which was the case for triglycerides, UAE and TMAO. TMAO values of 0.0 µM were replaced with 0.1 µM for statistical analysis purposes. Crude as well as age- and sex- adjusted Pearson correlation coefficients were calculated to explore relationships between clinical variables. Cox proportional hazards regression analyses were used to determine the risk for incident all-cause mortality according to quartiles of TMAO, as well as per 1 SD increment in TMAO. The assumption of proportional hazards for baseline predictors was investigated by inspecting the Schoenfeld residuals. Cox regression analyses with restricted cubic splines with three knots were carried out to show the association between TMAO and all-cause mortality. Multivariable analyses were conducted using Cox regression models including the covariates age, sex, BMI, smoking status, prevalent T2DM, history of CVD, history of cancer, use of anti-hypertensive medication, use of lipid lowering drugs, SBP, total cholesterol, HDL-C, triglycerides, eGFR, and UAE. Tests of trend across quartiles were conducted by assigning the median value for each quartile as its value and treating this as a continuous variable^32^. An advantage of this method is that is allows for use of all the intra-categorical information that is otherwise ignored by mere categorical comparisons. Possible effect modification was explored by including interaction terms between TMAO and age, sex, eGFR and HDL-C, in crude as well as in age- and sex-adjusted models. We carried out a mediation analysis to discern whether eGFR was a possible mediator between all-cause mortality and circulating TMAO levels following procedures outlined by Preacher and Hayes^33,34^. Covariates included age and sex. The bootstrapping technique advocated in this method was also employed. Cox proportional hazard regression analysis was also used to determine the risk for CVD. Interaction terms were considered to be statistically significant at two-sided P-values < 0.10^35^. Otherwise, the level of significance was set at two-sided P-values < 0.05. Given the enrichment of subjects with elevated UAE in PREVEND population, we also performed a secondary analysis in which we accounted for the sampling design of the study by using stratum-specific baseline hazard functions.

## Results

The clinical and laboratory characteristics of the 5,469 subjects are summarized in Table 1. The mean age of the study population was 53.5 ± 12.0 and the median TMAO level was 3.2 µM [1.71–5.70]. A history of CVD and T2DM were more prevalent among subjects in the highest quartile of TMAO. Levels of eGFR were lower in subjects in the highest quartile vs. subjects in the lowest TMAO quartile, whereas UAE >30 mg/24 h were more prevalent in subjects in the highest TMAO quartile.

**Table 1:** Baseline characteristics of the 5,469 subjects of the Prevention of Renal and Vascular End-Stage Disease (PREVEND) study according to quartiles of TMAO, µM.

	Quartiles of TMAO, µM				
	Q1 < 1.70	Q2 ≥ 1.71	Q3 ≥ 3.17	Q4 ≥ 5.70	P-value
Participants, n	1361	1373	1367	1368	
Age	48.7 ± 11.4	53.7 ± 11.7	55.5 ± 12.2	54.1 ± 12.0	<0.001
Sex, n (%)					<0.001
M	624 (45.8)	712 (51.9)	718 (52.5)	607 (44.4)	
F	737 (54.1)	661 (48.1)	649 (47.5)	761 (55.6)	
BMI, kg/m^2^	26.1 ± 4.3	26.5 ± 4.2	27.0 ± 4.4	27.1 ± 4.6	<0.001
Smoking status, n (%)					0.03
Non smoker	919 (67.5)	999 (73.0)	986 (72.1)	982 (71.8)	
Current smoker	415 (30.5)	359 (26.3)	363 (26.6)	374 (27.3)	
Hypertension, n (%)	374 (27.5)	450 (32.9)	516 (37.7)	471 (34.4)	0.003
Lipid lowering drug use, n (%)	106 (7.8)	119 (8.7)	156 (11.4)	142 (10.3)	0.005
History of CVD, n (%)	61 (4.5)	81 (5.9)	105 (7.7)	96 (7.0)	0.003
History of cancer, n (%)	67 (4.9)	81 (5.9)	75 (5.5)	89 (6.5)	0.33
T2DM, n (%)	53 (3.9)	67 (4.9)	96 (7.0)	120 (8.8)	<0.001
Blood pressure lowering drug use, n (%)	237 (17.4)	268 (19.5)	343 (25.1)	321 (23.5)	<0.001
Glucose lowering drug use, n (%)	30 (2.2)	40 (2.9)	57 (4.2)	72 (5.3)	<0.001
SBP, mmHg	123.5 ± 18.1	126.5 ± 18.7	128.0 ± 18.4	125.9 ± 19.8	<0.001
Total cholesterol, mmol/L	5.3 ± 1.0	5.5 ± 1.0	5.5 ± 1.1	5.4 ± 1.0	<0.001
Non-HDL cholesterol, mmol/L	4.1 ± 1.0	4.2 ± 1.0	4.2 ± 1.0	4.1 ± 1.0	<0.001
LDL cholesterol, mmol/L	1.29 ± 0.41	1.32 ± 0.41	1.31 ± 0.43	1.27 ± 0.41	0.005
HDL cholesterol, mmol/L	1.3 ± 0.3	1.3 ± 0.3	1.2 ± 0.3	1.2 ± 0.3	0.02
Triglycerides, mmol/L	1.05 [0.77–1.54]	1.15 [0.82–1.64]	1.18 [0.86–1.65]	1.11 [0.83–1.64]	<0.001
eGFR*crea-cysC*, mL/min/1.73 m^2^	96.7 ± 14.8	92.8 ± 15.9	90.2 ± 17.0	89.6 ± 19.5	<0.001
eGFR*crea-cysC*, mL/min/1.73 m^2^, categorical					<0.001
≥90	953 (70.0)	839 (61.3)	777 (56.8)	767 (56.1)	
≥60	392 (28.8)	504 (36.9)	524 (38.3)	499 (36.5)	
≥30	16 (1.2)	30 (2.2)	64 (4.7)	92 (6.7)	
<30	0 (0)	0 (0)	2 (0.1)	10 (0.7)	
UAE, mg/24 h	8.5 [5.9–14.0]	8.4 [6.0–37.9]	9.2 [6.3–17.4]	8.8 [6.0–18.1]	<0.001
UAE, mg/24 h, categorical					<0.001
>30	145 (10.7)	161 (11.8)	195 (14.3)	234 (17.2)	
≤30	1206 (89.3)	1207 (88.2)	1167 (85.7)	1126 (82.8)	

Data are numbers (percentages), means (SD) or medians [interquartile range (IQR)]. P-values were calculated by linear regression analysis or χ2 analysis. P-values were based on analysis of variance (nonskewed continuous variables), Kruskal–Wallis test (skewed continuous variables), or χ2 (categorical variables). Triglycerides, UAE and TMAO were logarithmically transformed for analysis. LDL was calculated in 5,372 subjects with fasting triglycerides <4.5 mmol/L. Abbreviations: BMI, body mass index; CVD, cardiovascular disease; eGFR*crea-cysC*, estimated glomerular filtration rate based on creatinine-cystatin C equation; LDL, low density lipoprotein; HDL, high density lipoprotein; SBP, systolic blood pressure; TMAO, trimethylamine N-oxide; T2DM, type 2 diabetes mellitus; UAE, urinary albumin excretion.

Pearson correlation coefficients between various clinical and laboratory variables and TMAO are given in Table 2. Univariable correlation analyses showed that age, BMI, hypertension, history of CVD, T2DM, SBP, triglycerides and UAE were positively associated with TMAO, whereas the female sex, current smoking, HDL-C and eGFR were inversely associated with TMAO. After adjustment for age and sex, TMAO was still positively correlated with age, BMI, T2DM and UAE, and inversely with current smoking, HDL cholesterol and eGFR.

**Table 2:** Pearson correlation coefficient between various clinical and laboratory variables and trimethylamine N-oxide (TMAO) (n = 5,469).

	TMAO, µM		Age- and sex-adjusted	
Clinical parameter	Pearson correlation	P-value	Pearson correlation	P-value
Age	**0.125**	**<0.001**	**0.125** ^1^	**<0.001**
Sex				
M	*Reference*		*Reference*	
F	**−0.027**	**0.046**	−0.016^2^	0.242
BMI, kg/m^2^	**0.09**	**<0.001**	**0.068**	**<0.001**
Smoking status				
Non smoker	*Reference*		*Reference*	
Current smoker	**−0.040**	**0.003**	**−0.030**	**0.032**
Hypertension	**0.061**	**<0.001**	0.007	0.668
History of CVD	**0.044**	**0.001**	0.025	0.115
T2DM	**0.082**	**<0.001**	**0.075**	**<0.001**
SBP, mmHG	**0.059**	**<0.001**	0.008	0.597
Total cholesterol, mmol/L	0.013	0.343	−0.022	0.159
HDL cholesterol, mmol/L	**−0.044**	**0.001**	**−0.041**	**0.009**
Triglycerides, mmol/L	**0.044**	**0.001**	0.022	0.158
eGFR*crea-cysC* (mL/min per 1.73 m^2^)	**−0.160**	**<0.001**	**−0.095**	**<0.001**
UAE, mg/24 h	**0.084**	**<0.001**	**0.062**	**<0.001**

Triglycerides, UAE and TMAO were logarithmically transformed for correlation analysis. ^1^Adjusted for sex. ^2^Adjusted for age. Abbreviations: BMI, body mass index; CVD, cardiovascular disease; eGFRcrea-cysC, estimated glomerular filtration rate based on creatinine-cystatin C equation; HDL, high density lipoproteins; SBP, systolic blood pressure; TMAO, trimethylamine N-oxide; T2DM, type 2 diabetes mellitus; UAE, urinary albumin excretion. Statistically significant correlations are shown in bold print.

**Figure 1:**
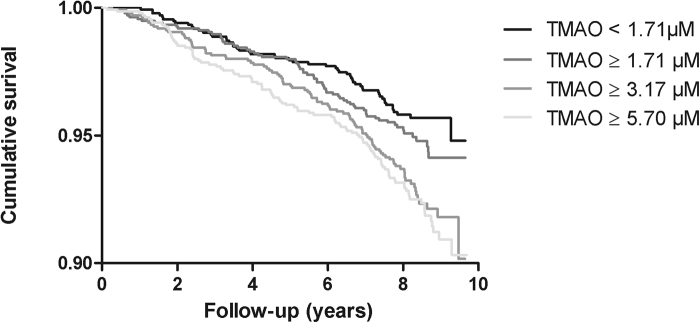
Kaplan-Meier curves of all-cause mortality according to quartiles of trimethylamine N-oxide (TMAO), P ≤ 0.001by log-rank test.

**Figure 2:**
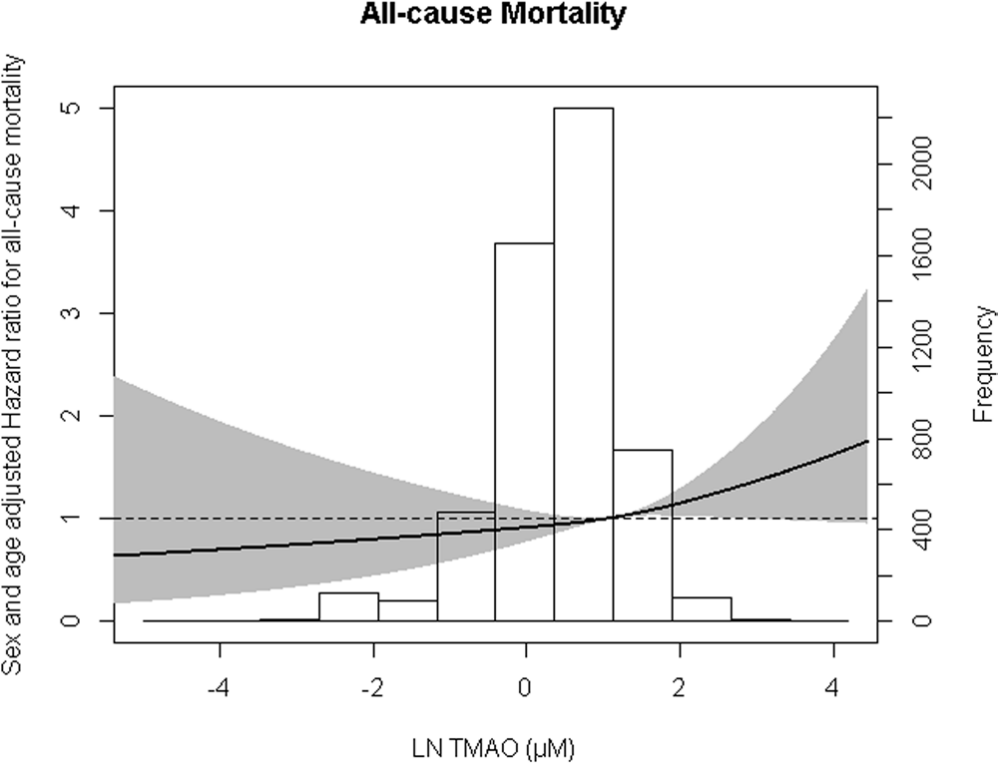
Association between trimethylamine N-oxide (TMAO) and all-cause mortality. Data were fit by a Cox proportional hazards regression model that was based on restricted cubic splines and adjusted for age and sex. The reference standard was mean TMAO level of 2.92 µM (antilog). The gray areas represent 95% CIs.

After a median follow-up period of 8.3 (7.8–8.8) years 322 (6.3%) subjects died. In unadjusted Cox regression models, higher plasma TMAO (the highest quartile) was associated with a 86% increased risk for all-cause mortality compared to the lowest quartile of TMAO (Table 3). Kaplan Meier curves are shown in Figs 1 and 2 shows an age- and sex- adjusted cubic spline for the association of TMAO with all-cause mortality. TMAO was independently associated with all-cause mortality in models adjusted for age, sex, BMI, smoking, T2DM, history of CVD, history of cancer, anti-hypertensive medications, lipid lowering drugs, SBP, total cholesterol, HDL-C and triglycerides (P for trend = 0.016, Table 3; model 1–3). The P-value for trend remained significant after adjustment for UAE (P = 0.049, model 4a). However, significance was lost after further adjustment for eGFR (P for trend = 0.15, model 4b). Comparable results were found in analysis with TMAO as a continuous variable, although statistical significance was lost after adjustment for UAE (model 4a). Furthermore, in crude, as well as in age- and sex-adjusted models and in models additionally adjusted for UAE, eGFR was associated with all-cause mortality (HR: 0.86, 95% CI: 0.75–0.98, P = 0.026 in age-, sex- and UAE-adjusted models).

**Table 3:** Association between trimethylamine N-oxide (TMAO) and all-cause mortality in 5,469 (322 cases) subjects of the Prevention of Renal and Vascular End-Stage Disease (PREVEND) study according to quartiles (Q1-Q4) and as continuous variable.

	**Q1**	**Q2**	P-value	**Q 3**	P-value	**Q 4**	P-value	P-value for trend	**TMAO per 1 SD increment**	P-Value
Subjects, n	1361	1373		1367		1368				
TMAO range, µM	<1.71	≥1.71–3.17		≥3.17–5.70		≥5.70				
No. of deaths	55	68		95		104				
Crude	(ref)	1.22 [0.86–1.75]	0.27	**1.71 [1.23–2.38]**	**0.002**	**1.86 [1.34–2.58]**	**<0.001**	**<0.001**	**1.28 [1.15–1.43]**	**<0.001**
Model 1	(ref)	0.98 [0.69–1.40]	0.90	1.05 [0.75–1.47]	0.76	1.37 [0.99–1.90]	0.06	**0.01**	**1.13 [1.01–1.27]**	**0.03**
Model 2	(ref)	1.00 [0.70–1.44]	0.99	1.11 [0.80–1.56]	0.53	**1.41 [1.01–1.96]**	**0.04**	**0.009**	**1.15 [1.03–1.29]**	**0.016**
Model 3	(ref)	0.96 [0.67–1.39]	0.85	1.06 [0.75–1.50]	0.72	1.36 [0.97–1.91]	0.07	**0.016**	**1.14 [1.01–1.28]**	**0.028**
Model 4a	(ref)	0.95 [0.66–1.38]	0.80	1.03 [0.73–1.46]	0.86	1.28 [0.91–1.80]	0.15	**0.049**	1.11 [0.99–1.25]	0.07
Model 4b	(ref)	0.93 [0.64–1.35]	0.71	1.00 [0.70–1.42]	0.98	1.20 [0.84–1.71]	0.32	0.15	1.08 [0.95–1.22]	0.23
Model 4c	(ref)	0.92 [0.64–1.34]	0.68	0.97 [0.68–1.38]	0.87	1.15 [0.81–1.64]	0.44	0.22	1.06 [0.94–1.20]	0.34

Hazard ratios and 95% confidence intervals were derived from Cox proportional hazards regression models. TMAO was logarithmically transformed before analysis. 1 SD change in TMAO corresponds to 2.94 µM (antilog). Model 1: age and sex. Model 2: Model 1 + body mass index and smoking. Model 3: Model 2 + type 2 diabetes mellitus, history of cardiovascular disease, history of cancer, anti-hypertensive medication, lipid lowering drugs, systolic blood pressure, total cholesterol, high density lipoprotein cholesterol and triglycerides. Model 4a: Model 3 + urinary albumin excretion (UAE). Model 4b: Model 3 + eGFR *crea-cysC* (estimated glomerular filtration rate based on creatinine-cystatin C equation). Model 4c: Model 3 + UAE and eGFR *crea-cysC*. Statistically significant correlations are shown in bold print.

The interaction terms between TMAO and age, sex, HDL-C and UAE were not significantly associated with all-cause mortality when included in the crude model (P for interaction >0.10 for all). Notably, however, the interaction term between TMAO and eGFR was significantly associated with all-cause mortality when included in crude and in the age- and sex-adjusted analyses (P for interaction 0.003 and 0.002, respectively).

Table 4 shows the Cox proportional hazards analysis stratified by eGFR (eGFR*crea-cysC* ≥90 and <90 mL/min per 1.73 m^2^). Median TMAO levels were higher in the group with impaired renal function compared to the group with normal renal function (Table 4, P < 0.001). In the group with eGFR*crea-cysC* ≥90 mL/min per 1.73 m^2^, TMAO was not significantly associated with all-cause mortality. Notably, in the group with eGFR <90, TMAO was significantly associated with all-cause mortality in crude analysis, as well as in models adjusted for age- and sex, and additionally for UAE.

**Table 4:** Association of trimethylamine N-oxide (TMAO) with all-cause mortality stratified by renal function (eGFR*crea-cysC*) in 5,469 (322 cases) subjects of the Prevention of Renal and Vascular End-Stage Disease (PREVEND) study.

eGFR*crea-cysC ≥*90 mL/min per 1.73 m^2^)		
Subjects (deaths)	3336 (103)	P-value
TMAO (µM)	2.91 [1.54–5.43]	
eGFR*creatcysC* (mL/min per 1.73 m^2^)	103.7 ± 9.0	
Crude	1.08 [0.91–1.30]	0.38
Model 1	0.99 [0.82–1.21]	0.97
Model 2	0.97 [0.80–1.18]	0.77
eGFR*crea-cysC <*90 mL/min per 1.73 m^2^)		
Subjects (deaths)	2133 (219)	P-value
TMAO (µM)	3.66 [2.04–6.11]	
eGFR*creatcysC* (mL/min per 1.73 m^2^)	76.0 ± 12.1	
Crude	**1.29 [1.12–1.48]**	**<0.001**
Model 1	**1.21 [1.05–1.39]**	**0.009**
Model 2	**1.18 [1.02–1.36]**	**0.023**

Median [IQR] TMAO levels are given. Hazard ratios and 95% confidence intervals were derived from Cox proportional hazards regression models. TMAO was logarithmically transformed before analysis. 1 SD change in TMAO corresponds to 2.94 µM (antilog). Model 1: age, sex. Model 2: Model 1 + UAE. Abbreviations: eGFR *crea-cysC*, estimated glomerular filtration rate based on creatinine-cystatin C equation; TMAO, trimethylamine N-oxide; UAE, urinary albumin excretion. Statistically significant correlations are shown in bold print.

In a secondary analysis, in which we accounted for the design of the PREVEND study with preferential inclusion of subjects with elevated UAE results remained essentially the same. The association between TMAO and all-cause mortality remained significant after adjustment for clinical covariates. Again, significance was lost when eGFR was included in the model (Supplemental Table 1).

**Figure 3:**
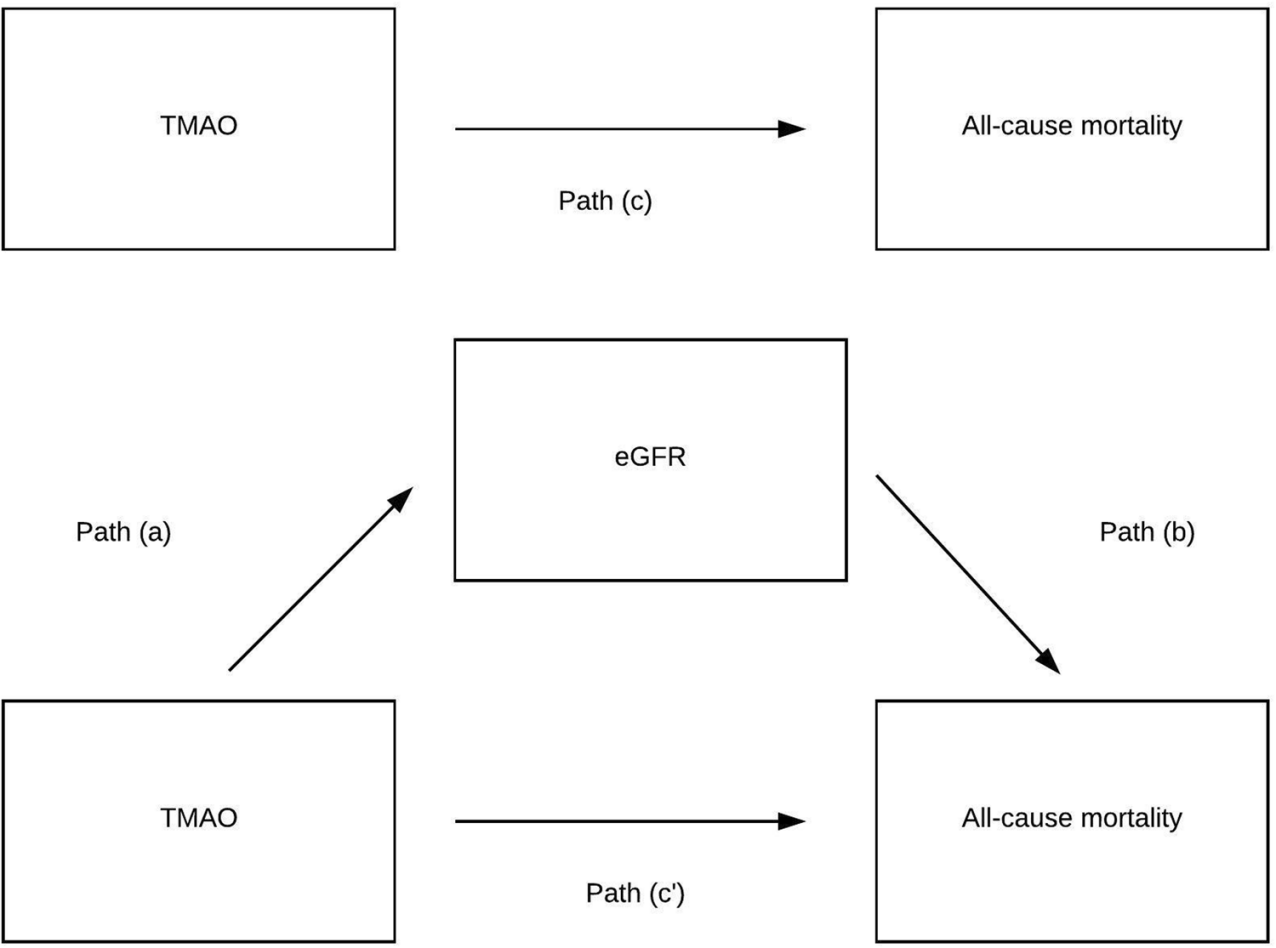
Mediation analysis on the association of TMAO with all-cause mortality. A, b and c are the standard regression coefficients between variables. The indirect effect is calculated as a * b. Total effect (c) is a * b + c’. Magnitude of mediation is calculated as indirect effect divided by total effect.

A mediation model is shown in Fig. 3. eGFR appeared to be a mediator in the association of TMAO with all-cause mortality. The indirect pathway was significant (*β* = 0.008, 95% CI: 0.002–0.016, *P*-indirect <0.005). The magnitude of mediation was 15% (Table 5).

**Table 5:** Mediating effect of eGFR on the association of trimethylamine N-oxide **(**TMAO) with all-cause mortality according to Preacher and Hayes Procedure.

	Coefficient (95% CI)*	Proportion mediated
Indirect pathway (*ab* path)	*β* = 0.008, 95% CI: 0.002–0.016	15%**
Total effect (*ab* + *c’* path)	*β* = 0.053, 95% CI: −0.016, 0.127	

Coefficients are adjusted for age and sex. *95% CIs were bias corrected confidence intervals after running 2000 bootstrap samples. **The size of the significant mediated effect is calculated as the standardized indirect effect divided by the standardized total effect multiplied by 100.

Supplemental Table 2 shows the results for TMAO and CVD. During follow-up 525 CVD events occurred. Because of the non-linear association between TMAO and CVD, results are presented with quartiles of TMAO. In crude as well as in and age- and sex- adjusted analysis TMAO in the highest quartile (≥5.7 µM) was significantly associated with CVD risk (P for trend <0.001 and 0.033). However, after multivariable adjustment, the P for trend was no longer significant (model 3).

## Discussion

This prospective study of 5,469 PREVEND study participants demonstrates that the microbiome-related metabolite, TMAO, is associated with all-cause mortality. Remarkably, the association of TMAO with mortality was attenuated after adjustment for albuminuria and became insignificant after adjustment for eGFR. In line, in subjects with any degree of decreased eGFR, TMAO was significantly associated with all-cause mortality even when taking account of albuminuria. In contrast, in subjects with normal renal function TMAO was not associated with mortality. Thus, a novel finding of our study is that the association of TMAO with mortality is modified by renal function in such a way that the association of TMAO with mortality is stronger in subjects with a lower eGFR.

While NMR was used in our study, other methods such as liquid chromatography tandem mass spectrometry (LC/MS-MS) are also available to quantify circulation TMAO levels^36–39^. Most of the clinical studies published using LC/MS-MS assays to date were conducted in populations of subjects at high CV risk with median TMAO levels ranging from 3.7–5.0 µM^4,6,10,16,40^. We sought to understand TMAO-disease associations in a population of subjects who were generally not at high CV risk, and whose median [interquartile range (IQR)] TMAO level was lower (3.2 [1.7–5.7] µM) than the previous studies. We did not observe an independent association between TMAO and CV outcomes in this population after adjustment for relevant covariates. This result appears to be in concert with other reports showing that TMAO may particularly predict (recurrent) CV events in high CV risk populations^2–6,8–14^. Of further interest, TMAO was lower in women but this difference was lost after controlling for age. In addition, TMAO was lower in subjects who were classified as current smokers at entry in the study. Lower TMAO levels in women could be attributable to sex differences in dietary habits such as lower intake of animal protein and lower egg consumption in women^41^. The association of TMAO with smoking may be explained by effects of smoking on intestinal microbiotica^42^, although this clearly needs further evaluation.

Early interrogation of the potential effects of diets, mimicking long-term exposure to elevated TMAO, in mice revealed that TMAO may directly contribute to progressive renal fibrosis and dysfunction^18^. Nonetheless, studies of the associations of TMAO with renal function and all-cause mortality in subjects with late-stage CKD have been mixed. One recent study reported that circulating TMAO predicted all-cause mortality in CKD patients who were candidates for renal transplantation^19^, while a second study reported that TMAO levels were elevated, but were not associated with all-cause mortality in patients new to dialysis^22^. Our study furthers this research by showing that TMAO is associated with all-cause mortality in subjects with any degree of renal function impairment, but not in subjects with normal renal function. Whether or not TMAO is a causative factor in the progression of renal or CV disease in humans deserves further study. However, it is clear from the current literature that TMAO is a prognostic marker, especially in subjects with CKD, independent of other CVD risk factors.

Recent reports have shown that circulating levels of TMAO are elevated in several chronic diseases such as obesity^43^, non-alcoholic fatty liver disease^44,45^, T2DM^12^ and heart failure^40,46^, thus raising the possibility that this microbiome-related metabolite could contribute to the increased CV risk in these often coexisting conditions. Of particular relevance, metabolites like TMAO are cleared by the kidney, and are therefore often linked to impaired renal function^17–19^. Elevated TMAO levels in subjects with an impaired renal function may be explained by higher production, reduced clearance or both^17^. Our study extends these earlier results by showing an inverse relationship of TMAO with eGFR in a large, well defined population-based cohort^22–24^. However, the potential mechanisms by which higher TMAO levels predict increased mortality risk, particularly in subjects with (mild to severe) renal function impairment remain incompletely understood. In an exploratory mediation analysis, set up to discern whether renal function makes part of the causal pathway of TMAO with mortality risk it was found that eGFR in part explained the association of TMAO with mortality. On the other hand, TMAO may directly contribute to progressive renal fibrosis and dysfunction in mice^18^, although equivocal associations of TMAO with renal function and all-cause mortality have been documented in humans with late-stage CKD^18,19^. Thus it is also possible that TMAO could contribute to renal function impairment which in turn is a determinant of mortality. Therefore, there may be a mutual cause and effect relationship between increased TMAO and chronic kidney disease adversely impacting on mortality^23,39^.

Several other methodological aspects of our study should be addressed. We consider the comprehensive assessment of laboratory variables, including lipoprotein fractions, eGFR and albuminuria in a large population of men and women a strength of our study. Notably, the PREVEND study population consists predominantly of Caucasians. Therefore, the applicability of the current results to other ethnic groups remains uncertain. Furthermore, as done in the present report, most epidemiological studies use a single baseline measurement for studying the association of variables with outcomes. Given the variability of TMAO over time^47^, this is anticipated to result in underestimation of the true strength of the association of this variable with outcome^48,49^. Further, the PREVEND cohort is enriched with subjects with elevated albuminuria. In the present report, the association of TMAO with outcome was only modestly modified by albuminuria, which itself is a determinant of worse outcome^50^. Moreover, in a secondary analysis, taking account of albuminuria enrichment in the cohort, a similar association of TMAO with mortality was observed which was again lost after further adjustment for eGFR. Finally, no information about dietary habits that could affect TMAO concentrations was available.

In conclusion, this prospective cohort study demonstrates that TMAO, a marker of the microbiome, is associated with all-cause mortality, independent of clinical and laboratory variables particularly in subjects with mild to severe renal function impairment.

## Electronic supplementary material

Supplementary information
