# Supplementary material for: TMAO is Associated with Mortality: Impact of Modestly Impaired Renal Function
Source: Sci Rep. 2017 Oct 23;7:13781. doi: 10.1038/s41598-017-13739-9 (PMC5653802; doi:10.1038/s41598-017-13739-9)
Supplement: Supplementary file 1 — Supplementary information [file 41598_2017_13739_MOESM1_ESM.pdf]

Supplementary Information

TMAO is Associated with Mortality: Impact of Modestly Impaired Renal Function

Eke G. Gruppen[a,b], Erwin Garcia[c], Margery A. Connelly[c], Elias J. Jeyarajah[c], James D. Otvos[c], Stephan J. L. Bakker[a], and Robin P.F. Dullaart[b]

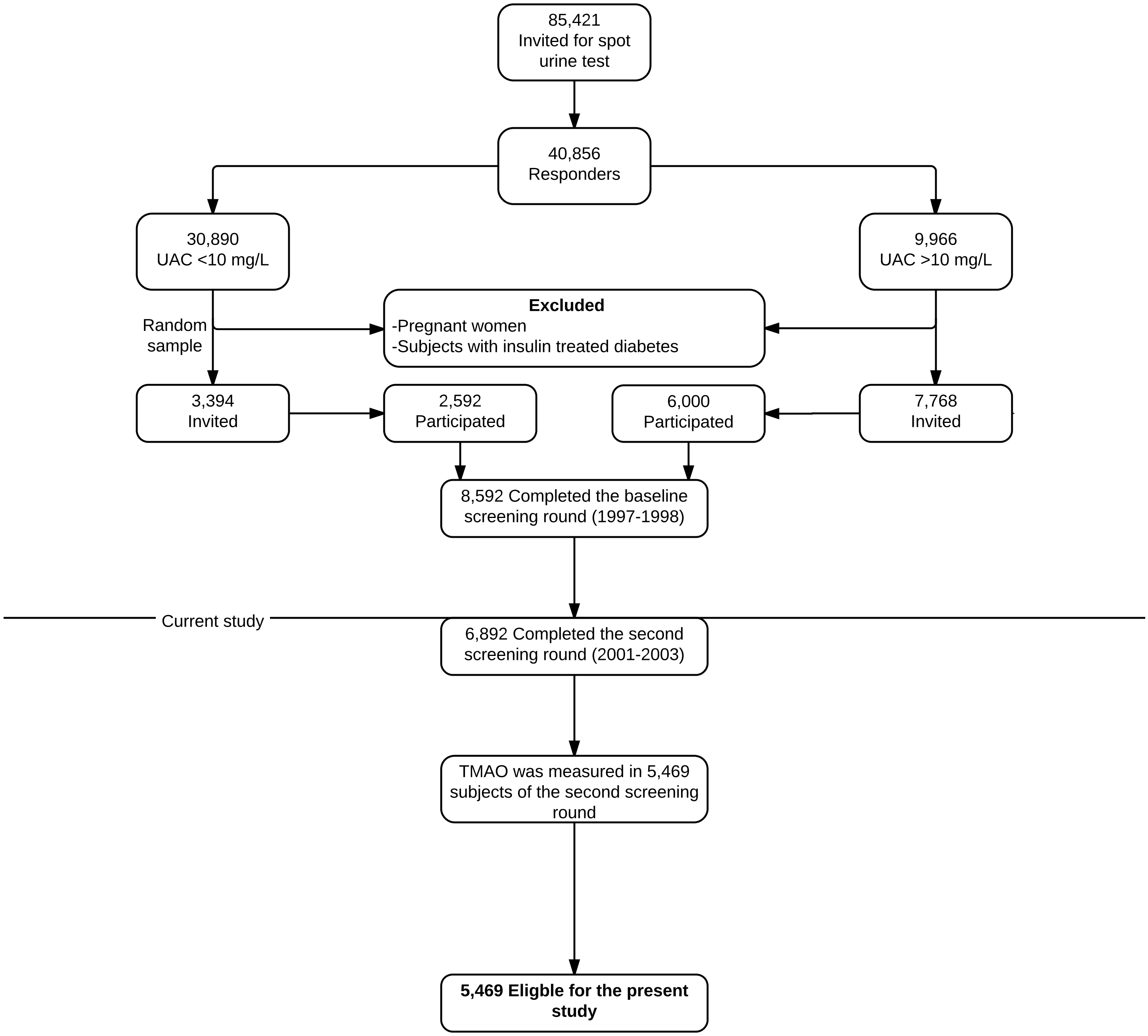

85,421
Invited for spot urine test
40,856
Responders
30,890
UAC <10 mg/L
9,966
UAC >10 mg/L
Excluded
-Pregnant women
-Subjects with insulin treated diabetes
Random sample
3,394
Invited
2,592
Participated
6,000
Participated
7,768
Invited
8,592 Completed the baseline screening round (1997-1998)
Current study
6,892 Completed the second screening round (2001-2003)
TMAO was measured in 5,469 subjects of the second screening round
5,469 Eligble for the present study

Supplemental Table 1. Association between trimethylamine N-oxide (TMAO) and all-cause mortality in 5,469 (322 cases) subjects of the Prevention of Renal and Vascular End-Stage Disease (PREVEND) study, accounting for the sampling design of the study (presence or absence of a urinary albumin concentration >10 mg/L) by specifying stratum-specific baseline hazard functions.

	**Q1**	**Q2**	P-value	**Q 3**	P-value	**Q 4**	P-value	P-value for trend	**TMAO per 1 SD increment**	P-value
Subjects, n	1361	1373		1367		1368				
TMAO range, µM	<1.71	≥1.71-3.17		≥3.17-5.70		≥5.70				
No. of deaths	55	68		95		104				
Crude	(ref)	1.21 [0.85-1.73]	0.29	**1.71 [1.23-2.39]**	**0.001**	**1.87 [1.35-2.60]**	**<0.001**	**<0.001**	**1.28 [1.15-1.43]**	**<0.001**
Model 1	(ref)	0.97 [0.68-1.38]	0.86	1.05 [0.75-1.46]	0.80	1.36 [0.98-1.89]	0.06	**0.014**	**1.13 [1.01-1.27]**	**0.03**
Model 2	(ref)	0.99 [0.69-1.42]	0.97	1.11 [0.79-1.55]	0.55	**1.41 [1.01-1.96]**	**0.04**	**0.010**	**1.15 [1.03-1.29]**	**0.015**
Model 3	(ref)	0.96 [0.66-1.39]	0.83	1.07 [0.75-1.51]	0.72	1.36 [0.97-1.91]	0.07	**0.017**	**1.14 [1.02-1.28]**	**0.027**
Model 4	(ref)	0.93 [0.64-1.35]	0.70	0.99 [0.70-1.42]	0.99	1.20 [0.84-1.71]	0.31	0.14	1.08 [0.95-1.22]	0.23

Hazard ratios and 95% confidence intervals were derived from Cox proportional hazards regression models. TMAO was logarithmically transformed before analysis. 1 SD change in TMAO corresponds to 2.94 µM (antilog).

Model 1: age and sex
Model 2: Model 1 + body mass index and smoking
Model 3: Model 2 + type 2 diabetes mellitus, history of cardiovascular disease, history of cancer, anti-hypertensive medication, lipid lowering drugs, systolic blood pressure, total cholesterol, high density lipoprotein cholesterol and triglycerides
Model 4: Model 3 + eGFR *crea-cysC* (estimated glomerular filtration rate based on creatinine-cystatin C equation)
Statistically significant correlations are shown in bold print.

Supplemental Table 2 Association between trimethylamine N-oxide (TMAO) and cardiovascular disease in 5,469 subjects (525 cases) of the Prevention of Renal and Vascular End-Stage Disease (PREVEND) study according to quartiles (Q1-Q4) and as continuous variable

	**Q1**	**Q2**	P-value	**Q 3**	P-value	**Q 4**	P-value	P-value for trend
Subjects, n	1361	1373		1367		1368		
TMAO range, µM	<1.71	≥1.71-3.17		≥3.17-5.70		≥5.70		
No. of cases	97	130		138		160		
Crude	(ref)	**1.45 [1.06-2.00]**	**0.022**	**1.51 [1.10-2.07]**	**0.011**	**1.79 [1.32-2.44]**	**<0.001**	**0.001**
Model 1	(ref)	1.21 [0.88-1.67]	0.24	1.00 [0.72-1.37]	0.98	**1.41 [1.03-1.92]**	**0.030**	**0.033**
Model 2	(ref)	1.22 [0.88-1.67]	0.23	1.02 [0.74-1.40]	0.93	**1.37 [1.00-1.86]**	**0.048**	0.06
Model 3	(ref)	1.16 [0.84-1.61]	0.36	0.96 [0.70-1.33]	0.81	1.24 [0.91-1.70]	0.18	0.22
Model 4a	(ref)	1.17 [0.85-1.62]	0.34	0.96 [0.69-1.33]	0.81	1.23 [0.89-1.69]	0.20	0.26
Model 4b	(ref)	1.07 [0.77-1.49]	0.68	0.87 [0.62-1.21]	0.41	1.09 [0.78-1.51]	0.61	0.62
Model 4c	(ref)	1.08 [0.77-1.50]	0.66	0.87 [0.62-1.22]	0.41	1.08 [0.78-1.50]	0.65	0.68

Hazard ratios and 95% confidence intervals were derived from Cox proportional hazards regression models. TMAO was logarithmically transformed before analysis. 1 SD change in TMAO corresponds to 2.94 µM (antilog).

Model 1: age and sex

Model 2: Model 1 + body mass index and smoking

Model 3: Model 2 + type 2 diabetes mellitus, history of cardiovascular disease, history of cancer, anti-hypertensive medication, lipid lowering drugs, systolic blood pressure, total cholesterol, high density lipoprotein cholesterol and triglycerides

Model 4a: Model 3 + urinary albumin excretion (UAE)

Model 4b: Model 3 + eGFR *crea-cysC* (estimated glomerular filtration rate based on creatinine-cystatin C equation)

Model 4c: Model 3 + UAE and eGFR *crea-cysC*

Statistically significant correlations are shown in bold print.
